# Proton Beam Therapy With Space-Making Surgery (Omental Plombage) for Oligorecurrent Liver Metastasis of Esophageal Adenocarcinoma

**DOI:** 10.7759/cureus.31656

**Published:** 2022-11-18

**Authors:** Yojiro Ishikawa, Koji Morita, Hisashi Yamaguchi, Takahiro Kato, Motohisa Suzuki, Ichiro Seto, Masanori Machida, Kanako Takayama, Takuya Tominaga, Yoshiaki Takagawa, Masanobu Nakajima, Yasushi Teranishi, Yasuhiro Kikuchi, Masao Murakami

**Affiliations:** 1 Department of Radiation Oncology, Southern Tohoku Proton Therapy Center, Koriyama, JPN; 2 Department of Radiology, Tohoku Medical and Pharmaceutical University, Sendai, JPN; 3 Department of Minimally Invasive Surgical and Medical Oncology, Fukushima Medical University, Fukushima, JPN; 4 Department of Radiation Physics and Technology, Southern Tohoku Proton Therapy Center, Koriyama, JPN; 5 Department of Medical Radiology Technology, School of Health Sciences, Fukushima Medical University, Fukushima, JPN; 6 First Department of Surgery, Dokkyo Medical University, Mibu, JPN; 7 Department of Surgery, Southern Tohoku General Hospital, Koriyama, JPN; 8 Department of Neurosurgery, Southern Tohoku Research Institute for Neuroscience, Southern Tohoku General Hospital, Koriyama, JPN

**Keywords:** oligometastasis, liver metastasis, esophageal adenocarcinoma, space-making surgery, proton beam radiotherapy

## Abstract

Proton beam therapy (PBT) with space-making surgery has been used recently; however, its effectiveness for recurrent esophageal cancer (EC) is unclear. We herein report an unusual case of successful PBT with space-making surgery (omental plombage) for recurrent liver metastasis after EC surgery. A 58-year-old Japanese man underwent proximal gastrectomy for esophagogastric (EG) junction cancer seven months before presentation to our hospital. Microscopic findings after the surgery showed that the tumor was adenocarcinoma of the EG junction (pT1N0M0, stage I). Seven months after the proximal gastrectomy, liver metastases in S6 and S8 were revealed by positron emission tomography-computed tomography. Initial PBT was performed for those two liver metastases, and complete response (CR) was obtained for both liver metastases. Recurrence of liver metastasis in S2 was found eight months after the first PBT, and CR was achieved by chemotherapy. However, new liver metastasis recurred in S2. Considering the effects of radiation exposure on the surrounding gastrointestinal organs, we performed space-making surgery to place the omentum around the liver metastasis. We were able to complete the second PBT for the liver metastasis with 72.6 Gy relative biological effectiveness in 22 fractions. After the second PBT, the patient survived for seven years without recurrence. PBT with space-making surgery (omental plombage) for recurrent liver metastasis after EC surgery is considered to be a therapeutic option.

## Introduction

Esophageal cancer (EC) is an aggressive disease and the recurrence rate is high [1,2]. EC often metastasizes to the liver after esophagectomy. Most patients with unresectable multiple liver metastases of EC can only receive systemic chemotherapy or palliative care, and their prognosis is usually poor. On the other hand, if the metastatic lesions are oligometastases, it is reasonable to consider performing local therapy, such as surgical resection, radiofrequency ablation (RFA), stereotactic radiotherapy (SRT) with photon beams, and particle ion therapy for a cure.

In the case of radiation therapy, a high dose of radiation might not be applicable due to the adverse effects on adjacent organs. In such cases, space-making surgery can reduce radiation exposure to the surrounding organs [3]. Furthermore, proton beam therapy (PBT) is considered to have less radiation exposure than photon therapy to the surrounding area due to the Bragg peak [4,5]. Therefore, PBT and space-making surgery may improve local control rates of liver metastases of EC.

In this case, it is reported that PBT and space-making surgery contributed to the control of oligorecurrent liver metastasis of esophageal adenocarcinoma (EAC). As far as we know, there has been no report of long-term survival in a patient with liver metastatic EAC.

## Case presentation

**Figure 1:**
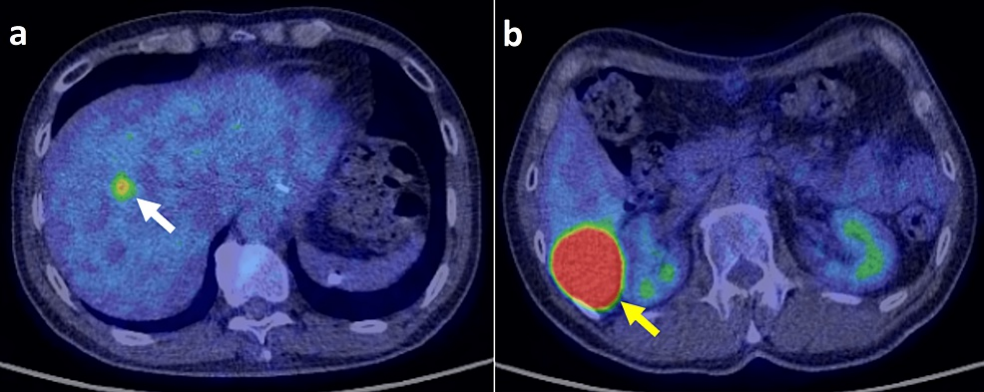
PET-CT before the first proton beam therapy. PET-CT showed two metastases in (a) S8 (10 mm) (white arrow) and (b) S6 (50 mm) (yellow arrow). There were no lymph node metastases or metastases at other sites.

A 58-year-old Japanese man underwent proximal gastrectomy for esophagogastric (EG) junction cancer seven months before presentation to our hospital. Microscopic findings after the surgery showed that the tumor was adenocarcinoma of the EG junction (pT1bN0M0, stage I). Seven months after the proximal gastrectomy with lymphadenectomy, liver metastases in S6 and S8 areas (measuring 50×40 mm and 10×10 mm in size, respectively) were revealed by positron emission tomography-computed tomography (PET-CT) (Figure 1, panels a and b).

The patient had a history of smoking (10 cigarettes per day for 38 years) and alcohol consumption (approximately 500 mL of beer per day for about 25 years). The patient had no medical history. Family history included colon cancer and liver cancer in his aunt and another aunt, respectively.

The surgeons recommended resection of the liver metastases. However, the patient refused to receive surgery due to concerns about the treatment side effects despite having an Eastern Cooperative Oncology Group Performance Score of 0. The surgeons also recommended chemotherapy; however, the patient refused to undergo chemotherapy due to concerns about the treatment outcome. He requested alternative treatment and was referred to our institution. We considered PBT, SRT with photon beams, or RFA for liver metastasis. However, we did not select SRT or RFA because the tumor size in S6 was over 50 mm.

**Figure 2:**
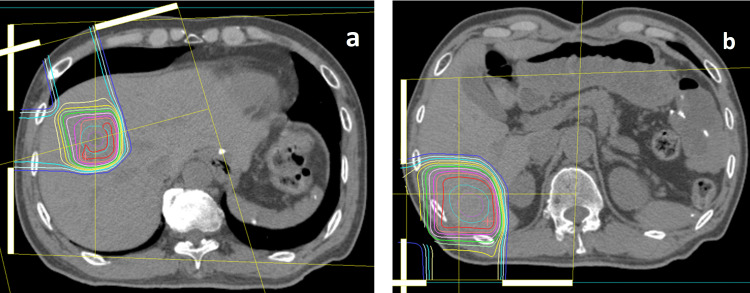
CT for the first proton beam therapy planning. The S8 (a) and S6 (b) lesions were treated by 66 GyRBE with 10 fractions for S8 tumor and 80 GyRBE with 25 fractions for S6 tumor. RBE: relative biological effectiveness

We recommended the combination of PBT and chemotherapy by IV administration; however, he and his family requested chemotherapy oral medication. We therefore performed PBT for liver metastases combined with S-1 (a combination of tegafur, gimeracil, and oteracil), 120 mg/day. The daily prescription dose of PBT was 2.4 and 6.6 Gy relative biological effectiveness (RBE) for liver metastases in S6 and S8, respectively. The S6 and S8 lesions received total doses of 80 and 60 GyRBE, respectively (Figure 2, panels a and b). The PBT system at our institute (Proton beam system; Mitsubishi, Tokyo, Japan) uses synchrotron and scattering methods. Treatment planning of PBT for liver lesions is based on three-dimensional CT images taken at 2 mm intervals in the exhalation phase while using a respiratory gating system (Anzai Medical, Tokyo, Japan). Treatment was administered during the exhalation phase using a respiratory gating system. Daily front and lateral x-ray imaging were used for positioning.

**Figure 3:**
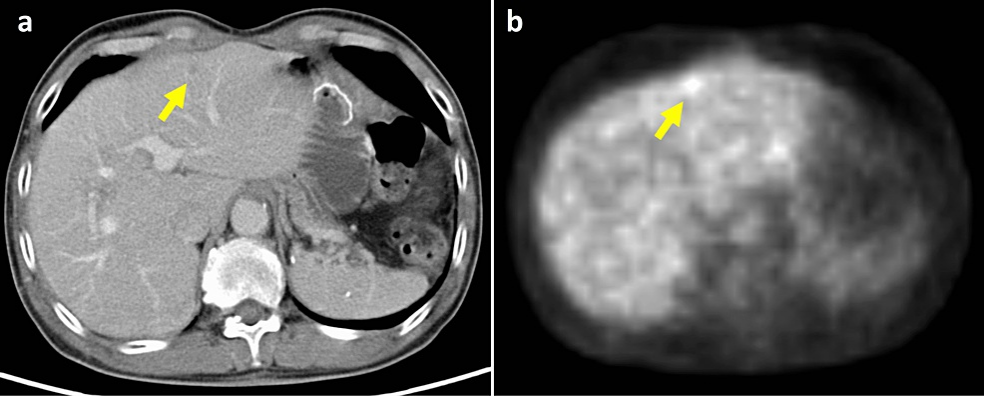
Contrast-enhanced CT and PET-CT one year after the first proton beam therapy. Contrast-enhanced CT (a) and PET-CT (b) one year after the first PBT. The liver metastasis was significantly regressed after four courses of docetaxel (70 mg/m^2^), cisplatin (70 mg/m^2^), and 5-FU (700 mg/m^2^) (yellow arrow). We could not rule out the possibility of metastasis from the EG junction cancer. EG: esophagogastric; PBT: proton beam therapy; 5-FU: 5-fluorouracil

Although the liver metastases disappeared, PET-CT showed another liver metastasis in S2 one year after the first PBT (Figure 3, panels a and b). The surgeons recommended systemic chemotherapy, and four courses of docetaxel (70 mg/m^2^), cisplatin (70 mg/m^2^), and 5-fluorouracil (5-FU) (700 mg/m^2^) were carried out. The liver metastases were significantly regressed after the chemotherapy.

**Figure 4:**
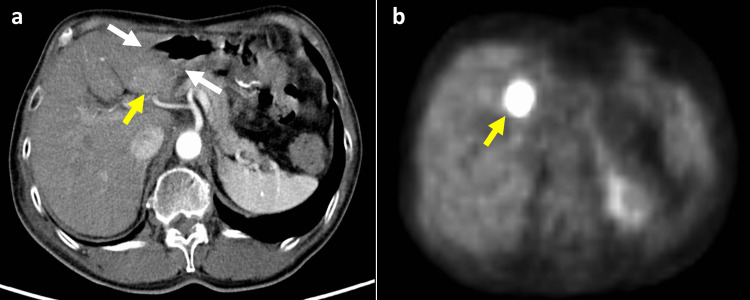
Contrast-enhanced CT and PET-CT after four courses of chemotherapy. Contrast-enhanced CT (a) and PET-CT (b) after four courses of chemotherapy showed new liver metastasis in the S2 liver (yellow arrow). This recurrence lesion was located adjacent to the gastrointestinal tract (white arrow).

**Figure 5:**
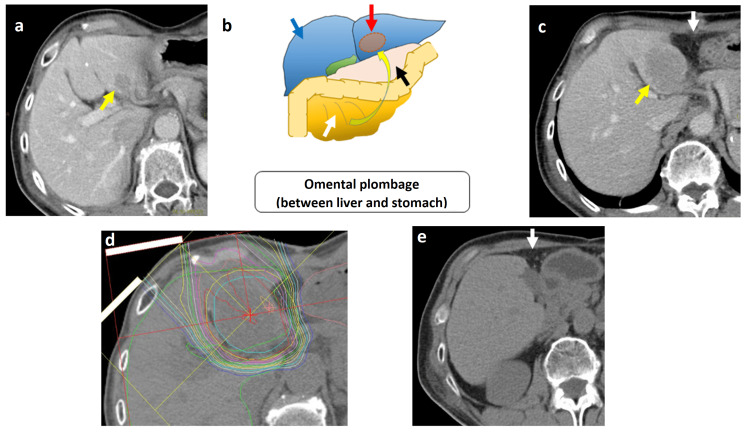
Timeline of proton beam therapy with space-making surgery (omental plombage) for oligorecurrent liver metastasis of esophageal adenocarcinoma. Contrast-enhanced CT showed the lesion in S2 (yellow arrow) (a). The liver metastasis in S2 was very close to adjacent organs including the esophagus and the remaining stomach, and we therefore decided to perform the space-making surgery using the remaining omentum after the proximal gastrectomy. An image of space-making surgery using the omental plombage is shown. Blue, white, red, and black arrows indicate the liver, great omentum, tumor, and stomach, respectively (b). After space-making surgery, CT showed that the omentum (white arrow) was located between the liver metastasis in S2 (yellow arrow) and stomach (c). The second PBT for the liver metastasis in the S2 was performed with 72.6 GyRBE in 22 fractions (d). Seven years after the second PBT, a complete response was maintained and the inserted omentum has still remained (e).

After the four courses of chemotherapy, PET-CT and CT showed another liver metastasis (measuring 40×40 mm in S2) (Figure 4, panels a and b; Figure 5, panel a). The surgeons judged that the chemotherapy had failed. The patient had a third metastatic recurrence; however, it was considered to be an oligorecurrence due to solitary liver metastasis. However, resection was not selected due to concerns about the treatment outcome of liver resection and its side effects. The patient requested PBT again and was referred to our institution. PBT alone was not acceptable since the recurrence tumor in the liver was located broadly adjacent to the gastrointestinal tract. Therefore, we decided to perform a novel two-step treatment with surgical spacer placement and subsequent PBT.

Space-keeping surgery was performed by open abdominal surgery. The gastric lesser curvature and the inferior surface of the liver had strong postoperative adhesions. After detaching the postoperative adhesions, the lesion of the liver S3 was identified and the space between the gastric kyphosis and the undersurface of the liver was filled with omentum. No pathology specimen was taken from the tumor of the liver S3 (Figure 5, panels b and c). Two weeks after the spacer surgery, we performed PBT for the S2 liver metastasis combined with S-1 (120 mg/day).

The gross tumor volume (GTV) included liver metastasis in S2 and was 37.27 cm^3^. The clinical target volume (CTV) was defined as GTV plus 0.5 cm margins. The planning target volume (PTV) was CTV plus 0.5 cm margins. The CTV and the PTV were 78.30 and 137.42 cm^3^, respectively. The PTV received total doses of 72.6 GyRBE in 22 fractions. The patient received adjuvant chemotherapy (two courses of DCF) (Figure 5, panel d).

After PBT, an acute side effect of grade 1 dermatitis according to the National Cancer Institute Common Terminology Criteria for Adverse Events version 5.0. occurred, but there was no acute or late complication of more than grade 2. CT and PET-CT after treatment showed no evidence of recurrence. The patient is doing well seven years after the second PBT as well as nine years after the first PBT (Figure 5, panel e).

## Discussion

EC is a complex disease to treat. After radical oesophagectomy, the liver recurrence rate of EC is 6-25%, and the prognosis after recurrence is poor [6-8]. Patients with multiple metastatic EC can usually receive only systemic chemotherapy or palliative therapy, and their five-year survival rate is less than 5% [9]. However, if the number of metastases is small, additional operations, SRT and RFA are also treatment options. Van Daele et al. reported that the overall survival rate and disease-free survival rate after surgery for metastatic esophageal cancer were 50% and 33%, respectively. In general, metastatic patients who can undergo hepatic resection tend to have a better prognosis [10]. Gandy et al. reported a five-year survival rate of 60% after hepatic resection for non-colorectal, non-endocrine liver metastases [11].

In recent years, oligometastases or oligorecurrence have been attracting attention. Cancer status with less than five metastatic or recurrent lesions and primary controlled lesions can be considered "oligorecurrence" [12]. A previous study showed that chemoradiotherapy could cure patients' oligorecurrence in the lymph nodes of EC [8].

In addition to these treatments, PBT has been increasingly used in recent years. PBT is a treatment method with Bragg-peak characteristics and a higher dose-concentration system than that of conventional photon therapy, reducing exposure to normal tissues [4,5]. The five-year overall survival rates for EC of stages I, II, III, and IV were 79.3%, 66.3%, 43.2%, and 28.3%, respectively. The three- and five-year local control rates were 70.2% and 64.4%, respectively [13]. However, there have been few reports on recurrent EC. Recently one report also reported a case of long-term survival after PBT for postoperative oligometastasis and recurrence of EC [14]. However, even with the physical characteristics of PBT, it is difficult to treat tumors that are near the gastrointestinal tract. Particle therapy with space-making surgery has been reported in gynecological cancers, pancreatic body and tail cancers, abdominal leiomyosarcoma, and abdominopelvic sarcoma in close proximity to the gastrointestinal tract [15-17]. The liver is an organ with a gastrointestinal tract in close proximity, and particle therapy in combination with space-making surgery is being attempted. Komatsu et al. reported the two-year overall survival rate was 39.1% in 17 patients of combination treatment with surgical spacer placement and subsequent particle radiotherapy for unresectable hepatocellular carcinoma [18]. Hashimoto et al. also reported the treatment outcomes and effectiveness of the 12 unresectable hilar cholangiocarcinomas (HCs) treated with space-making surgery. The one- and three-year overall survival rates of HCs were 82.5% and 45.8%, respectively [19]. As far as we know, there has been no report of treatment of liver metastasis after recurrence of EC using a spacer.

**Figure 6:**
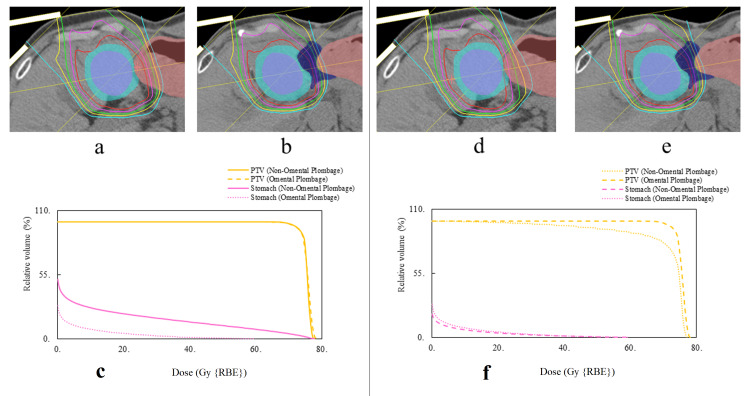
Simulation plans of dose distribution and dose-volume histogram for proton beam therapy without space-making surgery and proton beam therapy with space-making surgery. CT images showed that GTV, PTV, stomach, and omental plombage are indicated by purple area, right blue area, red area, and dark blue area, respectively. Assuming that PBT was performed without spacer surgery, the treatment plan was simulated by CT before spacer treatment, and D95%, D98%, and D2cc of OAR were compared using DVH. We divided the plan without space-making surgery (omental plombage) into a plan where the tumor was irradiated with priority and a plan where the priority was to reduce the dose to the stomach. Dose distributions of PBT with priority given to PTV dose without space-making surgery (omental plombage) (a) and dose distribution after space-making surgery (omental plombage) (b). In DVH, the dose to the stomach is reduced after space-making surgery (omental plombage) (c). Dose distributions of PBT with priority on reducing the dose to the stomach without space-making surgery (omental plombage) (d) and after space-making surgery (omental plombage) (e). In DVH, the dose to the PTV after space-making surgery (omental plombage) is clearly increased (f). DVH: dose-volume histogram; PBT: proton beam therapy; GTV: gross tumor volume; PTV: planning target volume; OAR: organ at risk

In our case, we performed PBT using an omentum spacer to treat liver metastases adjacent to the gastrointestinal tract after EC surgery. We compared the dose distributions before and after the spacer surgery. The dose distribution was better after the spacer surgery (Figure 6, panels a-f).

Computed tomography after the spacer showed that the distance between the tumors and stomach was 11 mm or more. In DVH, the tumor coverage was good (D95 = 72.6 GyRBE, D98 = 70.4 GyRBE), and it was kept within the tolerable dose of D2cc = 43.5 GyRBE in the adjacent stomach. Radiation dermatitis grade 1 was the only acute adverse event, and more than seven years have passed since the second PBT, but there are no late adverse events and the patient has not relapsed. Assuming that PBT was performed without spacer surgery, setting D95% = 72.6 GyRBE for the tumor would result in D2cc = 75.8 GyRBE for the stomach, and setting D2cc = 43.5 GyRBE for the stomach would result in D95% = 44.2 and D98% = 29.2 GyRBE for the tumor. It was found that spacer placement is essential for the safe and reliable proton therapy of tumors adjacent to the gastrointestinal tract.

Space-making surgery can be achieved by implanting an artificial object or an omentum. In the present case, there was a possibility that a part of the omentum had been treated during the initial EC surgery. Therefore, implantation of an artificial object was an option, but treatment could be safely performed with the remaining omentum. It is believed that the risk of infection is lower with an omentum spacer than with an artificial spacer, and this may be one of the reasons for the safe treatment in this case. Still, it is not easy to define the superiority of the material of the spacer in this case.

## Conclusions

PBT for oligo-liver metastasis can be a curative treatment. When an organ at risk (OAR) is in close proximity, spacer surgery can reduce the OAR dose and irradiate the tumor with a sufficient dose. Because this study was a case study, it is difficult to define the indication for PBT with space-making surgery for recurrent liver metastasis after EC surgery. However, it is possible that some patients with recurrent liver metastasis after EC surgery were treated only by surgery, chemotherapy, or radiation therapy despite being potential candidates for PBT with space-making surgery. PBT with space-making surgery for recurrent liver metastasis after EC surgery is considered to be a therapeutic option.
